# Global surveillance of emerging SARS-CoV-2 variants

**DOI:** 10.2471/BLT.22.289528

**Published:** 2024-06-01

**Authors:** Mahmut Uludağ, Roberto Incitti, Xin Gao, Jonathan L Heeney, Takashi Gojobori, Intikhab Alam

**Affiliations:** a Computational Biology Research Center, 4700 King Abdullah University of Science and Technology, Thuwal 23955-6900, Makkah, Saudi Arabia.; b Department of Veterinary Medicine, University of Cambridge, Cambridge, England.

Variants of the severe acute respiratory syndrome coronavirus 2 (SARS-CoV-2) such as Alpha, Beta, Delta and the highly mutated Omicron lineage have caused waves of infections following worldwide spread of the original virus since 2020.^1^ Multiple variants continue to evolve, with thousands of infections reported globally each week. The continued surveillance of emerging SARS-CoV-2 variants is therefore needed, especially considering the threat of several regionally emerging sublineages of Omicron such as the hypermutated (ab.100 mutations) BA.2.86 and JN that have immune evasion properties. A study reported that BA.2.86 is resistant to class 2 and 3 monoclonal antibodies directed to epitopes in receptor-binding domain as well as having a high receptor affinity, approximately twofold compared to XBB.1.5 or EG.5.1. The new subvariant of BA.2.86, JN.1, exceeds 100 mutations compared to wild type, suggestive of an evolutionary detour.^2^

The World Health Organization (WHO) provides guidance on tools for surveillance of SARS-CoV-2 based on scientific data from genomic sequencing.^3^ These global data are essential to understand the virus’ evolution, such as the frequency of variants of interest and the signalling of variants of concern. The coronavirus disease 2019 (COVID-19) Virus Mutation Tracking platform^4^^,^^5^ is included among these tools.

We originally developed the platform to allow advanced analyses of SARS-CoV-2 genomes from the Global Initiative on Sharing All Influenza Data (GISAID). Updated weekly, the platform provides worldwide geographic surveillance of variants and mutations based on a data volume of over 16 million SARS-CoV-2 genomes to date. The platform also includes geographic sequencing as well as tracking of vaccination efforts, providing a timely report on how many variants of unique copies of SARS-CoV-2 genomes have been reported.

Because new variants emerge and continue to evolve before they fit the defined nomenclature, the platform provides an approach based on virus-defined amino acid mutation fingerprints, augmented with variant labels from WHO, GISAID and Pango lineages.^6^ An interactive table on the platform presents the mutation fingerprints. This table, which is of interest to the scientific and medical community, can be queried interactively for several attributes.^7^

For example, considering the BA2.86 and JN variants, the platform shows more than 6000 mutation fingerprints supported by at least three complete SARS-CoV-2 genomes, with the related trajectory of isolates across countries for one of the mutation fingerprints. As another example, when querying the mutation fingerprints table for entries associated with at least 10 000 isolates, we identify 26 mutation fingerprints. These fingerprints represent the most spreading genomes of SARS-CoV-2, providing important information for health authorities to develop appropriate interventions, including vaccine development.

To track emerging mutations, the platform provides a free-access variant detection tool for rapid analyses, where users can input partial or complete SARS-CoV-2 genomes. The information generated provides the geographic distribution and frequency of immune-evading mutations, as well as the key mutations unique to a variant or shared among variants that show evidence of being variants of concern. Thus, this set of key mutations with related mutation fingerprints provide further detailed analyses to support evidence-based public health decisions on measures to combat new variants, as well as develop pan-variant vaccine candidates and therapeutic antibodies. Such vaccine could address the increasing global health concerns on immune-evading mutations of SARS-CoV-2.^2^^,^^8^

While it is not possible to forecast which new variant will become a variant of concern,^9^ the platform tracks emergence and frequency of variants over time. However, doing so requires continued representative sequencing of SARS-CoV-2 genomes in immunocompromised, vulnerable populations, as well as in healthy vaccinated and non-vaccinated individuals globally, as WHO suggests.^10^ Notably, the mutation rate of variants of concern is relatively higher for a brief period after they are first detected. This rate typically declines over time as population immunity builds. Mutation rate analyses can be explored further based on the mutation fingerprints interactive table in the platform.

While current databases only track mutations occurring in the virus, monitoring the animal reservoirs and related coronaviruses remains important. Lastly, identification of conserved epitopes across the broader sarbeco^8^ and merbeco coronaviruses may lead to the identification of important targets for more broadly protective Beta-coronavirus vaccines.
